# Measles Epidemiology in Ethiopia from 2006 - 2016: Predictors of High Measles Incidence from Surveillance Data Analysis

**DOI:** 10.29245/2578-3009/2018/si.1118

**Published:** 2018-09-01

**Authors:** Teklay K Desta, Ephrem T. Lemango, Jimma D Wayess, Balcha G Masresha

**Affiliations:** 1 Maternal and Child Health Directorate, FMOH Ethiopia, P.O. Box 1234, Addis Ababa, Ethiopia; 2 Ethiopian Public Health Institute, FMOH. P.O. Box 1242, Addis Ababa, Ethiopia; 3 World Health Organization, Regional Office for Africa, Brazzaville, Congo

**Keywords:** Ethiopia, Epidemiology, Elimination, Incidence, Measles, Surveillance

## Abstract

**Background:**

Ethiopia endorsed the African Regional measles elimination goal and has been implementing the recommended strategies. Measles immunization coverage has been increasing but is still below the target, and measles incidence has remained high.

**Objective:**

To describe the measles epidemiology in Ethiopia, identify predictors of high measles incidence in Ethiopia and recommend strategies to achieve the elimination goal.

**Methods:**

Measles surveillance 2006-2016 data, routine immunization and post measles campaign coverage data was analyzed. We analysed the epidemiology and incidence of measles cases by age, vaccination status, year of occurrence, and geographic area.

**Result:**

There were 66,719 confirmed cases, out of the 94,104 suspected measles cases reported between January 2006 and December 2016. Measles incidence increased from 20 cases per million total population in 2006 to 194 cases per million in 2015 and declined to 49 per million in 2016. On multiple logistic regression analysis, the median age of measles cases, the 2013 measles Supplemental Immunisation Activity (SIAs) coverage, the 2012 routine immunization coverage, and the proportion of reported under-five measles cases were predictors of very high measles incidence (>240 cases per million in the under-five years age population) in the three-year period following the 2013 measles SIAs implementation (p<0.01).

**Conclusion:**

Ethiopia is not on track to achieve the measles elimination goal of less than 1 case per million population by 2020 with the current pace of elimination efforts. Accumulation of susceptible children due to suboptimal routine measles immunization combined with suboptimal and narrow age–group (9-59 months) measles SIAs resulted in continued measles outbreaks.

**Recommendation:**

Ethiopia should scale up the quality and implementation of all the measles elimination strategies, including the introduction of measles second dose and conducting high quality measles SIAs targeting the appropriate age groups as per the measles epidemiology in various parts of the country to accelerate and achieve the 2020 measles elimination goal.

## Introduction

Measles is one of the vaccine preventable diseases among the leading causes of under-five child mortality in Ethiopia^1^. The Expanded Program on Immunisation (EPI) was started in Ethiopia in 1980 with six antigens including measles vaccine. The measles vaccination coverage has been increasing over the past years in Ethiopia. The national immunization coverage survey in 2012 revealed routine measles immunization coverage of 68.2% in children 12-23 months of age^2^, and measles vaccination coverage according to the WHO/UNICEF Estimates of National Immunization Coverage (WUENIC) was 78% in 2015^3^.

In 2001, countries in the African region adopted the measles mortality reduction strategy. The recommended strategies for measles mortality reduction include providing the first dose of measles vaccination (MCV1) at or shortly after 9 months of age through routine services and a second dose of measles vaccine through either routine service (MCV2) or through Supplemental Immunization Activities (SIAs)^4,5,6^. Following the implementation of these strategies, an estimated 60% reduction in global measles mortality was achieved by 2005^7,8^. During 2000–2008, global mortality attributed to measles declined by 78%, from an estimated 733,000 deaths in 2000 to 164,000 in 2008, while estimated measles mortality declined by 92% in the African region^9^.

In September 2011, the Sixtieth Regional Committee of the World Health Organisation (WHO), by its Resolution AFR/RC61/R1, adopted a goal of measles elimination for the African Region by the year 2020 (10). In 2012, Ethiopia endorsed the measles elimination 2012-2020 goal and has been implementing the recommended strategies^11,12,13^. The national measles elimination strategic plan set the following objectives to be achieved by 2020: achieve and maintain measles incidence <1 cases per million populations, achieve and maintain MCV1 coverage >95% at National and District levels and at least ≥ 95% SIAs coverage in all Districts, and introduction of a measles 2^nd^ dose in the routine immunisation schedule^11^. However, as of June 2017, the second dose of measles vaccination in routine immunisation services (MCV2), has not yet been introduced in the immunization schedule in Ethiopia. By the end of 2015, the African Region has documented 85% reduction in measles deaths as compared to measles mortality estimates for the year 2000^14^.

Ethiopia implemented the initial measles catch-up Supplemental Immunization Activities (SIAs) in 2002-2003 and several follow up SIAs have been conducted so far. In June 2013, measles immunization follow-up SIAs was conducted nationwide, targeting children between 9-59 months of age. The post SIA coverage survey result showed national coverage of 90.7%^15^. Paradoxically, the number of measles cases increased by two fold and three fold in 2014 and 2015 respectively following the 2013 measles SIAs^16^. In April 2016, wide-age range measles SIAs (targeting children aged 6 - 179 months old) was conducted in 545 districts in Ethiopia.

Measles surveillance is the detection, reporting and investigation of measles cases. Measles case based surveillance has been in place in Ethiopia since 2003, and is supplemented by laboratory confirmation of suspected cases starting from 2004^17,18^.

Ethiopia has documented an increase in reported measles cases from 3,332 in 2002 (the year of the first sub-national catch-up measles SIAs) to 17,745 in 2015^19^. This increase in cases is attributed to improved sensitivity of surveillance, but also due to outbreaks of confirmed measles occurring in different parts of the country^20^.

The investigation and response to measles outbreaks detracts attention and resources from efforts to strengthen immunisation systems. It has been documented that measles outbreaks in Ethiopia incur economic costs amounting to US$72.29/case for the Health sector, (including outbreak response immunization campaign), and US$29.18/case for households, equal to 6% of the household median annual income^21^. The objective of this study is to describe the measles epidemiology in Ethiopia, identify predictors of high measles incidence and recommend strategies and interventions to achieve the 2020 measles elimination goal.

## Methods

Measles case-based surveillance is coordinated by the Ethiopian Public Health Institute (EPHI). In this case based surveillance system, suspected each measles case is investigated and reported using a standard case reporting form, and blood specimen is collected to test for measles immunoglobulin M(IgM). The measles case based surveillance data includes standard epidemiological variables like the unique identification number, age, sex, district of residence, vaccine doses received, IgM test results, final classification of cases and outcome. Measles surveillance data from the years 2006 - 2016 was analysed alongside the post-campaign coverage survey data from the SIAs in 2013, and the data from the regional routine immunization coverage survey in 2012. Data analysis was done using SPSS 20 software. The surveillance data was recoded to create categorical variables such as age groups (confirmed measles cases under 5 years of age, above 5 years of age), vaccination status (vaccinated, not vaccinated), and period in relation to measles 2013 SIAs. The proportion of measles cases aged less than five years old, and the median age of measles cases was calculated for each woreda. Laboratory confirmed, clinically compatible, and epidemiologically linked measles cases were classified as confirmed measles cases as defined in the national measles surveillance and outbreak management guideline^22^. Measles incidence, was computed for the different age groups, regions, zones and woredas.

Univariate and multiple logistic regression analysis was done to determine the association between woreda measles incidence in under five-year old children (dependent variable) and the explanatory variables (routine immunization coverage survey result from 2012, post measles SIAs coverage result after the 2013 SIAs, median age of measles cases, population density, geographic area, and the proportion of confirmed under-five measles cases) to determine predictors of high measles incidence. P value of < .05 was considered statistically significant. Step wise logistic regression with backward elimination was performed to identify predictors of highest measles incidence (>240 cases per million under-five population, which corresponds to the 75^th^ percentile of the distribution of measles incidence by woreda level). The significance level for including independent variables in the model was P<0.1 and p<0.05 for dropping from the model.

The following operational definitions were used.

**Confirmed measles cases**: suspected measles cases that were notified to the surveillance system and confirmed by IgM laboratory serological testing, or by epidemiological linkage or clinical compatibility.

**Vaccinated measles cases**: Measles cases who have received one or more measles vaccine doses were classified as “vaccinated cases” and those with zero doses and unknown status were classified as “not vaccinated”.

**Measles incidence**: measles incidence was calculated by dividing the confirmed measles cases by population-year, and then multiplied by one million to compute measles incidence per million population for single or multiple year period. Source of total population was from CSA population projection.

**Proportion of vaccinated measles cases**: The number of vaccinated cases as defined above divided by total measles cases.

**Proportion of measles cases in the under five-year age group**: the proportion of under five-year old measles cases out of the total confirmed measles cases.

## Results

### Epidemiological description of reported measles cases

**Figure 1:**
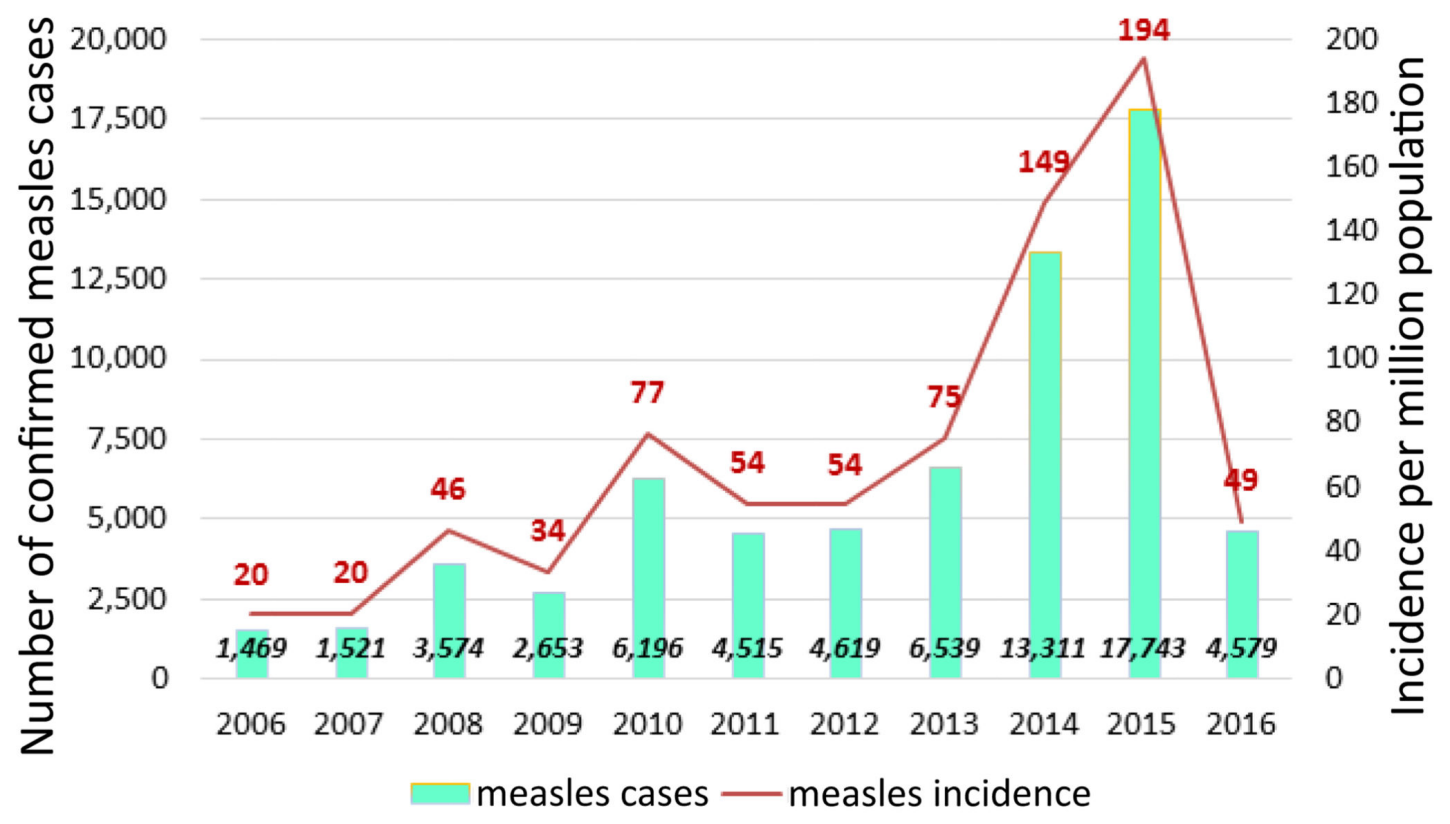
Distribution of confirmed measles cases and annual measles incidence per million total population by year. 2006 - 2016. Ethiopia.

There were 66,719 (70.9%) confirmed cases, out of the 94,104 suspected measles cases reported from January 2006 to December 2016. Measles incidence increased from 20 cases per million in 2006 to 194 cases per million population in 2015 and declined to 49 per million in 2016 following the wide-age group measles SIAs. The number of confirmed measles cases was highest in 2015 with 17,743 confirmed cases (Figure 1).

The proportion of confirmed measles cases aged less than 5 years was 42% and the median age of confirmed measles cases was 6 years for the period 2006 – 2016. The median age of measles cases ranges from 3 years in Gambella to 15 years in Tigray Region (Table 1).

**Figure 2:**
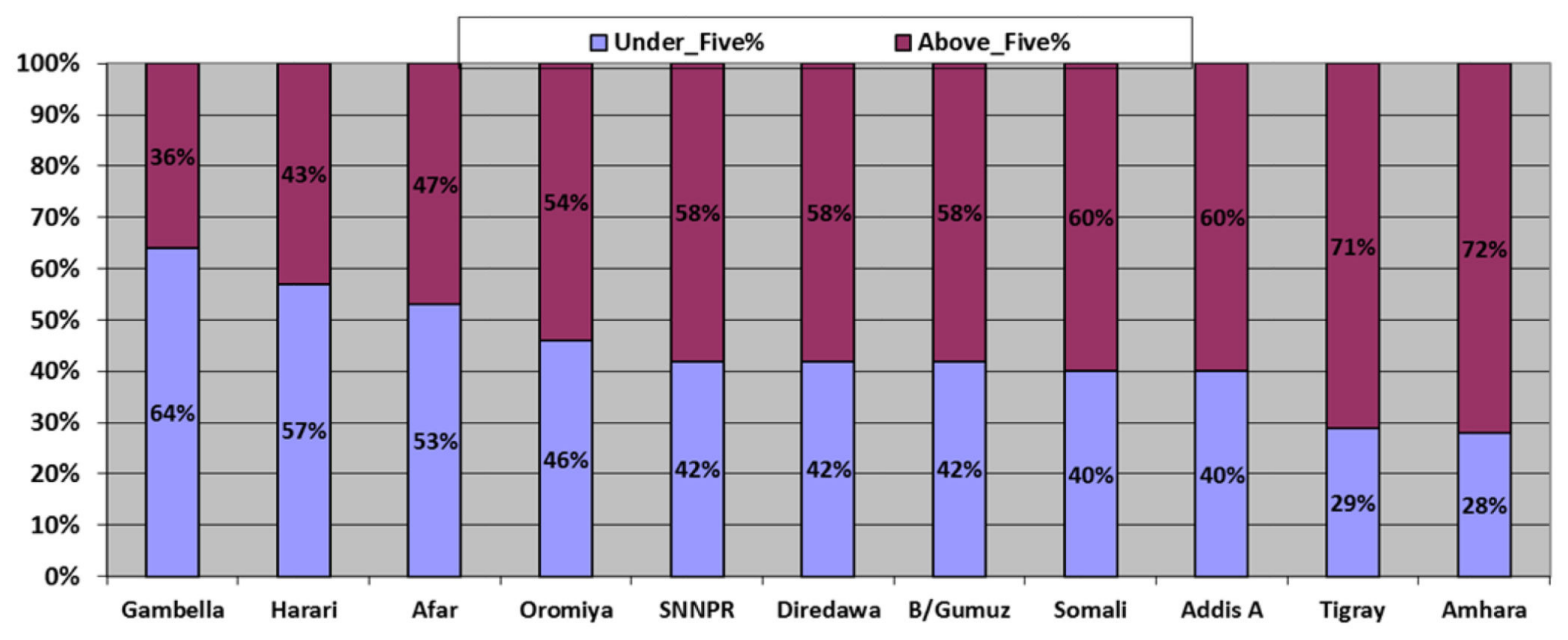
Proportion of under-five and above-five years old conformed measles cases by region for the 2006-2016 period, Ethiopia

**Figure 3:**
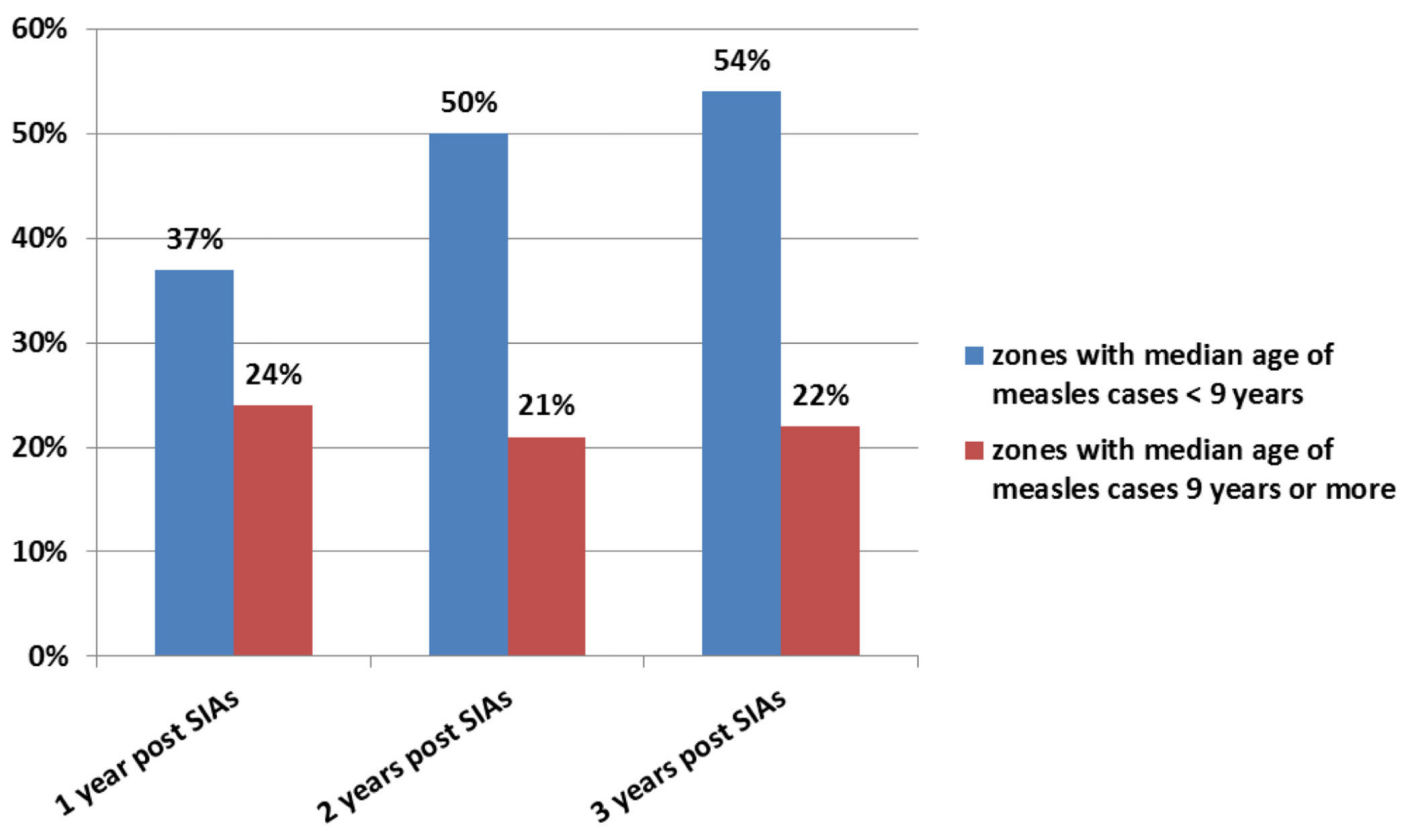
Proportion of under-five measles cases in the years following 2013 measles SIAs in Zones with median age below 9 years and those 9 years and above.

The proportion of under-five cases was high for developing regions such as Gambella (64%), and Afar (53.0%) (Figure 2). On the other-hand, Amhara and Tigray Regions have relatively low proportion of under-five cases (less than 30%). Within the same Region, the zonal proportion of under-five cases vary. In zones with median age lower than 9 years, there was a rapid buildup of under-five measles cases in the three years following the measles SIAs conducted in 2013 as depicted by high and increasing under-five year proportion (37%, 50% and 54% respectively for the three consecutive years post campaign). Conversely, zones with median age 9 years or older had stable proportion of under-five cases (24%,21% and 22%) for the three years following the 2013 measles SIAs (Figure 3). The proportion of confirmed measles cases who had received at least one dose of measles vaccine was 26% and 31% for all age groups and under-five old children respectively for the 2006 - 2016 period.

#### Factors accounting for high number of measles cases following measles SIAs 2013

Regions with more than 80% measles routine immunization coverage as per the result of the 2012 survey had an average measles incidence in 2012 of 91 cases per million as compared to Regions with less than 80% survey coverage (198 per million population). Woredas located in regions with more than 80% measles routine immunization survey coverage had significantly lower incidence of measles in children under-five (less than 240 cases per million population) in the three years (July 2013-June 2016) period following the 2013 measles SIAs. The proportion of woredas located in Regions with routine measles coverage above 80% that had lower measles incidence was higher than those woredas in Regions with less than 80% measles coverage (86% vs 71%, p=X^2^ = 3.96, P=0.047).

**Figure 4:**
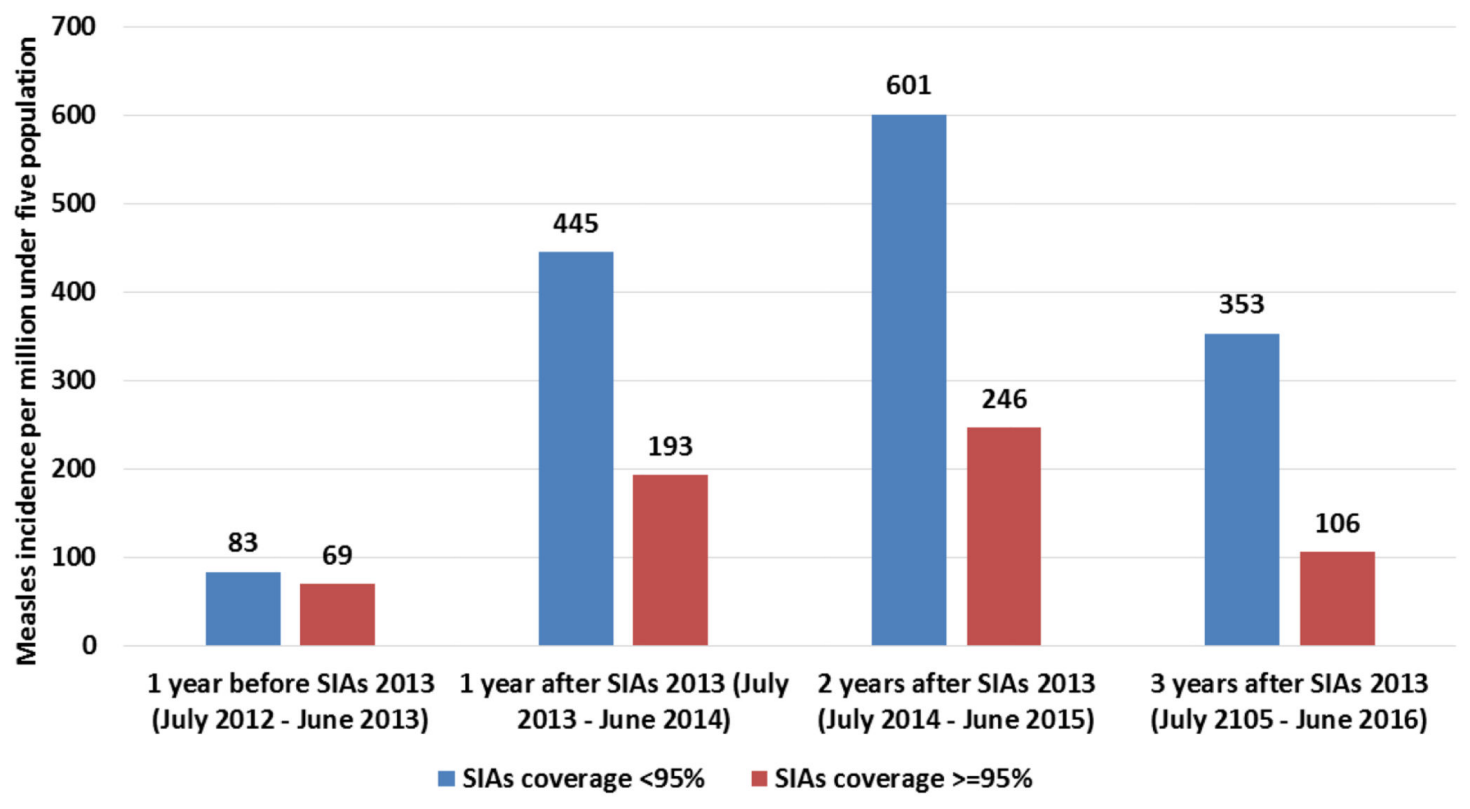
Measles incidence per million under-five population, in the years after the 2013 measles SIAs in zones with different post-measles SIAs survey coverage levels (<95% vs >=95%).

There was lower measles incidence in under five years old children in zones with post measles SIAs coverage of 95% and above compared to those zones with less than 95%, in the three years following the 2013 measles SIAs (Figure 4). A large proportion of woredas located in zones with post measles SIAs immunization coverage of 95% and above had lower incidence of measles in the under-five population (less than 240 cases per million population) compared to those woredas located in zones with less than 95% coverage (82.9% vs 71.0%, X2,12.79, p<0.001) in the three years period following measles SIAs 2013.

The proportion of measles cases in the age group under-five was significantly associated with measles incidence. The proportion of woredas with highest incidence of measles in the under-five year old population (Incidence >240 per million) was 10.6%, 21.3%, 31.6% and 39.7% for woredas with proportion of under five cases <=24%, >24%-<36%, 36%-50% and >50% respectively (X^2^ = 45.3, p<0.001). Similarly, the proportion of woredas with highest under-five year measles incidence (>240) was 37.3%, 33.8%, 17.9% and 11.8% for those woredas with median age of < 4 years, 4-6 years, >6-10 years and > 10 years respectively (X^2^ = 44.9, p<0.001).

### Predictors of high measles incidence

On univariate analysis, proportion of under-five year cases, median age of measles cases, routine and SIAs immunization coverage, population density were statistically associated with measles incidence (Table 2). The median age of measles cases, routine and SIAs immunization coverage were inversely correlated with measles incidence whereas proportion of under-five cases and population density was positively correlated with under-five year measles incidence.

On multiple logistic regression analysis, the median age of measles cases, the coverage result reported from the post measles SIAs coverage survey, the routine immunization coverage results from the survey in 2012, and the proportion of under-five cases were predictors of highest measles incidence (>240 cases per million under-five years age population) in the three-year period following the 2013 measles SIAs implementation (p<0.01) (Table 3). The predicting power of the model was 74%.

## Discussion

Measles under-five mortality is estimated to have decreased from 716 per 100,000 in 1990 to 87 per 100,000 in 2013 in Ethiopia^23^. However, according to the case based surveillance data, measles incidence in Ethiopia has been increasing in the last ten years. Partly, this could be due to improved reporting system and confirmation of suspected cases. The annualized rate of investigation of suspected measles cases per 100,000 increased from 2.9 in 2006 to 4.8 in 2015 at the national level^18,24^. However, there was an unprecedented increase in the number of measles cases in 2014 and 2015 following the measles SIAs in 2013.

The possible reasons for continued high incidence are: 1) low routine immunization coverage (measles vaccination coverage of 68.2% according to the 2012 survey report), 2) measles second dose has not yet been introduced in the routine immunization system, 3) the proportion of children receiving measles vaccine before 9 months age (receiving invalid measles doses) is high, estimated at 24% nationally in 2012 (25), 4) suboptimal measles SIAs coverage, with post SIA coverage survey result of 90.9% for the 2013 measles SIAs, which is below the 95% coverage target for SIAs (25), 5) SIAs conducted between 2006-2015 period targeted children under the age of 5 years only, (which account for only 42% of the cases), while the epidemiological pattern of measles was gradually shifting to older age groups, hence outbreaks continued to be propagated by susceptible older children accumulated over the years. This is substantiated by the fact that only 6% of the 13,311 cases in 2014 were under five-year old children who reside in zones with >=95% coverage according to the post-measles SIAs coverage survey in 2013. Among the remaining, 67% cases were older children that were not targeted in the SIAs. On the other hand, 27% of the cases in zones with inadequate coverage (<95%) were aged under five years.

Measles SIAs targeting children under-five have limited and transient impact on reducing measles incidence and preventing outbreaks in many African countries^26^. An assessment conducted to measure the impact of nationwide measles SIAs conducted in 1999-2001 for children 6 months to 5 years in Uganda revealed that measles incidence declined by 39%, and measles deaths by 63% in the year following the campaigns, with impact lasting 15 to 22 months^26^.

Measles incidence normally decreases following measles campaigns. In Vietnam the incidence of measles was reduced from 5.44 in 2001 to 0.14 per 100,000 in 2002 following a national measles campaign which achieved 99% coverage^27^. However, without high coverage with two doses of measles vaccine in the routine program, resurgence of measles outbreak is common in countries with single dose schedule even after SIAs achieving high coverage. In Vietnam, despite high MCV1 coverage which surpassed 93% since 1993 with a one-dose schedule, measles outbreaks occurred every 7-8 years, indicating the limitation of a single dose approach to interrupt circulation of measles virus^27^. Hence the introduction of a measles second dose in the routine immunization program is an important programmatic opportunity to build population immunity, and fast track the progress towards measles elimination.

This study showed that Regions and zones in Ethiopia with high median age of confirmed measles cases, which have low proportion of under-five cases have overall lower rate of measles incidence compared to those with low median age of measles cases. The epidemiological shift of measles susceptibility towards older age groups is one of the features that follow a reduction in the intensity of measles transmission^28^. Measles case based surveillance from 40 African countries for the period of 2002-2009 showed that the median age of the confirmed measles cases was significantly associated with the measles vaccination coverage in the countries. The median age for all of the confirmed cases was 3 years. However, the percentage of cases in the 9-59 months age group decreased as measles coverage increased^29^. Similarly, in France, the proportion of measles cases of ages over 10 years increased from 13 % in 1985 to 48% in 1997 following an increase in the immunization coverage from 32% in 1985 to over 80% in 1994-9 at 24 months of age^30^. This is consistent with our study where regions with higher median age of measles cases have low under-five proportions and relatively lower measles incidence compared to those with low median age of measles cases. In this regard, Tigray and Amhara have low proportion of under-five measles cases (29% and 28% respectively), high median age of cases (14 and 9 years respectively) and low measles incidence for the 2006 - 2016 period (50 and 70 per million population per year respectively). On the contrary, Oromia and SNNPR Regions have lower median age of measles cases (4 and 5 years respectively), high proportion of measles cases in the under-five age group (46% and 42% respectively) and higher measles incidence per million population year (130 and 132 respectively).

The incidence of measles in Ethiopia is high and has remained above 5 per 1,000,000 for the past five years. This is above the target set for accelerated measles control less than 5 per 1,000,000 for 2015^31,32^ or measles elimination target, less than 1 per 1,000,000. One reason for the recurrent measles outbreaks could be early vaccination of children in the routine immunization schedule. The proportion of invalid measles dose (children vaccinated for measles in routine immunization before 9 months age) in Ethiopia was reported to be 26.3%^33,34^. Combined with the low level of routine immunization coverage (78%, WUENIC, 2015), this can leave more than one third of the children in each cohort susceptible for measles.

The midterm review of the implementation of measles elimination strategies in Ethiopia conducted in October 2016 has underscored the delay in the progress towards measles elimination by 2020. The review assessed the progress towards the measles elimination target as compared to the set targets by WHO AFRO using the presented data for each strategy and concluded that the progress is off track^35^.

## Conclusion

Ethiopia is not on track to achieve the measles elimination goal of less than 1 case per million population by 2020 with the current pace of elimination efforts. Accumulation of susceptible children due to suboptimal routine measles immunization, combined with suboptimal and narrow age scope measles SIAs resulted in continued measles outbreaks. Ethiopia should implement all the measles elimination strategies including the introduction of measles second dose to accelerate and achieve the goal of measles elimination. In the coming years, Ethiopia should implement high quality follow-up measles SIAs with age targets tailored according to the regional epidemiology in order to close the growing immunity gap in children aged 5 – 9 years of age and older. Zones and regions with extreme age shift in measles susceptibility need to extend the target age group of SIAs beyond the usual age of 9 months to 15 years.

## Figures and Tables

**Table 1:** Distribution of measles cases by region, Ethiopia, 2006-2016.

Region	Suspected measles cases (2006 - 2016)	Confirmed measles cases**	Median age of confirmed measles cases (years)	# (%) of confirmed measles cases under 5 years age	measles incidence per million population
Addis Ababa	4,780	1,869	6	719 (40%)	70
Afar	1,124	819	3	433 (53%)	36
Amhara	14,324	10,212	9	2,820 (28%)	70
B/Gumuz	1,268	821	6	341 (42%)	139
Dire Dawa	238	115	6	48 (42%)	38
Gambella	846	770	2	492 (64%)	327
Hareri	647	196	3	112 (57%)	77
Oromia	41,191	31,802	4	14,528 (46%)	130
SNNPR	23,477	17,077	5	7,117 (42%)	132
Somali	1,639	1,366	5	541 (42%)	26
Tigray	2,927	1,672	14	495 (30%)	50
Grand Total	92,461	65,865	5	27,646 (42%)	102

** confirmed cases refers to cases confirmed by laboratory (IgM positive), epidemiological linkage or clinical compatible cases.

**Table 2:** Explanatory variables associated with high/low measles incidence on univariate analysis.

Variable	Categories	Total Woredas	Measles Incidence in under-5 year age within 3 years following the 2013 measles SIAs (Incidence per million population)		X^2^, p values
			<=240 per million	>240 per million	
Median age of measles cases	< 4 years	177	111(62.7%)	66(37.3%)	X^2^=44.9, P<0.0001
	4.0-6.0 years	198	131(66.2%	67(33.8%)	
	6.1-10.0 years	168	138(82.1%)	30(17.9%)	
	> 10 years	195	172(88.2%)	23(11.8%)	
Proportion of under-five measles cases among all confirmed cases	<=24%	188	168(89.4%)	20(10.6%)	X^2^=45.3, P<0.0001
	>24-<36%	169	133(78.7%)	36(21.3%)	
	36-50%	193	132(68.4%)	61(31.6%)	
	>50%	174	105(60.3%)	69(39.7%)	
2012 Measles routine immunization coverage by survey (2012)	<=80%	687	509(74.1%)	178(25.9%)	X^2^ =3.96, p=0.047
	>80%	57	49(86.0%)	8(14.0%)	
2013 measles SIAs coverage by survey	<=95%	458	325(71.0%)	133(29.0%)	X^2^ =12.79, p=0.0001
	95-100%	263	218(82.9%)	45(17.1%)	
Area	Urban	12	9(75.0%)	3(25.0%)	X^2^ =0.2, p=0.735
	Agrarian	625	472(75.5%)	153(24.5%)	
	Pastoral	107	77(72.0%)	30(28.0%)	
Population density people per Km^2^	<=30	280	179(63.9%)	101(36.1%)	X^2^ =31.59, p<0.0001
	31-60	144	115(79.9%)	29(20.1%)	
	61-90	147	116(78.9%)	31(21.1%)	
	91-400	173	148(85.5%)	25(14.5%)	

**Table 3:** Predictors of highest measles incidence (>240 per million in under-five year children) using multiple logistic regression.

Under-five years measles incidence (cut off point 240 per million population)	B	Odds Ratio	Std. Err.	P	[95% confidence Interval]	
Measles RI coverage (2012 survey)	0.0197	1.019931	0.008153	0.014	1.004076	1.036037
Post-Measles SIAs survey coverage (2013)	0.0352	1.035861	0.01353	0.007	1.009679	1.062722
Median age of measles cases	0.0738	1.07659	0.039164	0.042	1.002502	1.156153
Proportion of measles cases aged under five years	-0.0165	0.983654	0.008186	0.048	0.96774	0.999831
Constant	-3.3299	0.035798	0.050202	0.018	0.002292	0.559183
